# Evolutionary history of anglerfishes (Teleostei: Lophiiformes): a mitogenomic perspective

**DOI:** 10.1186/1471-2148-10-58

**Published:** 2010-02-23

**Authors:** Masaki Miya, Theodore W Pietsch, James W Orr, Rachel J Arnold, Takashi P Satoh, Andrew M Shedlock, Hsuan-Ching Ho, Mitsuomi Shimazaki, Mamoru Yabe, Mutsumi Nishida

**Affiliations:** 1 Natural History Museum and Institute, Chiba, 955-2 Aoba-cho, Chuo-ku, Chiba 260-8682, Japan; 2 School of Aquatic and Fishery Sciences, College of Ocean and Fishery Sciences, University of Washington, Campus Box 355020, Seattle, WA 98195-5020, USA; 3 National Marine Fisheries Service, Alaska Fisheries Science Center, 7600 Sand Point Way NE, Seattle, WA 98115, USA; 4 Collection Center, National Museum of Nature and Science, 3-23-1 Hyakunincho, Shinjuku-ku, Tokyo 169-0073, Japan; 5 Department of Organismic and Evolutionary Biology, Museum of Comparative Zoology, Harvard University, 26 Oxford Street, Cambridge, MA 02138, USA; 6 Institute of Marine Biology, National Taiwan Ocean University, 2 Peining Road, Keelung 202, Taiwan; 7 Laboratory of Marine Biodiversity, Graduate School of Fisheries Sciences, Hokkaido University, 3-1-1 Minato-cho, Hakodate, Hokkaido 041-8611, Japan; 8 Ocean Research Institute, The University of Tokyo, 1-15-1 Minamidai, Nakano-ku, Tokyo 164-8689, Japan

## Abstract

**Background:**

The teleost order Lophiiformes, commonly known as the anglerfishes, contains a diverse array of marine fishes, ranging from benthic shallow-water dwellers to highly modified deep-sea midwater species. They comprise 321 living species placed in 68 genera, 18 families and 5 suborders, but approximately half of the species diversity is occupied by deep-sea ceratioids distributed among 11 families. The evolutionary origins of such remarkable habitat and species diversity, however, remain elusive because of the lack of fresh material for a majority of the deep-sea ceratioids and incompleteness of the fossil record across all of the Lophiiformes. To obtain a comprehensive picture of the phylogeny and evolutionary history of the anglerfishes, we assembled whole mitochondrial genome (mitogenome) sequences from 39 lophiiforms (33 newly determined during this study) representing all five suborders and 17 of the 18 families. Sequences of 77 higher teleosts including the 39 lophiiform sequences were unambiguously aligned and subjected to phylogenetic analysis and divergence time estimation.

**Results:**

Partitioned maximum likelihood analysis confidently recovered monophyly for all of the higher taxa (including the order itself) with the exception of the Thaumatichthyidae (*Lasiognathus *was deeply nested within the Oneirodidae). The mitogenomic trees strongly support the most basal and an apical position of the Lophioidei and a clade comprising Chaunacoidei + Ceratioidei, respectively, although alternative phylogenetic positions of the remaining two suborders (Antennarioidei and Ogcocephaloidei) with respect to the above two lineages are statistically indistinguishable. While morphology-based intra-subordinal relationships for relatively shallow, benthic dwellers (Lophioidei, Antennarioidei, Ogcocephaloidei, Chaunacoidei) are either congruent with or statistically indistinguishable from the present mitogenomic tree, those of the principally deep-sea midwater dwellers (Ceratioidei) cannot be reconciled with the molecular phylogeny. A relaxed molecular-clock Bayesian analysis of the divergence times suggests that all of the subordinal diversifications have occurred during a relatively short time period between 100 and 130 Myr ago (early to mid Cretaceous).

**Conclusions:**

The mitogenomic analyses revealed previously unappreciated phylogenetic relationships among the lophiiform suborders and ceratioid familes. Although the latter relationships cannot be reconciled with the earlier hypotheses based on morphology, we found that simple exclusion of the reductive or simplified characters can alleviate some of the conflict. The acquisition of novel features, such as male dwarfism, bioluminescent lures, and unique reproductive modes allowed the deep-sea ceratioids to diversify rapidly in a largely unexploited, food-poor bathypelagic zone (200-2000 m depth) relative to the other lophiiforms occurring in shallow coastal areas.

## Background

**Figure 1:**
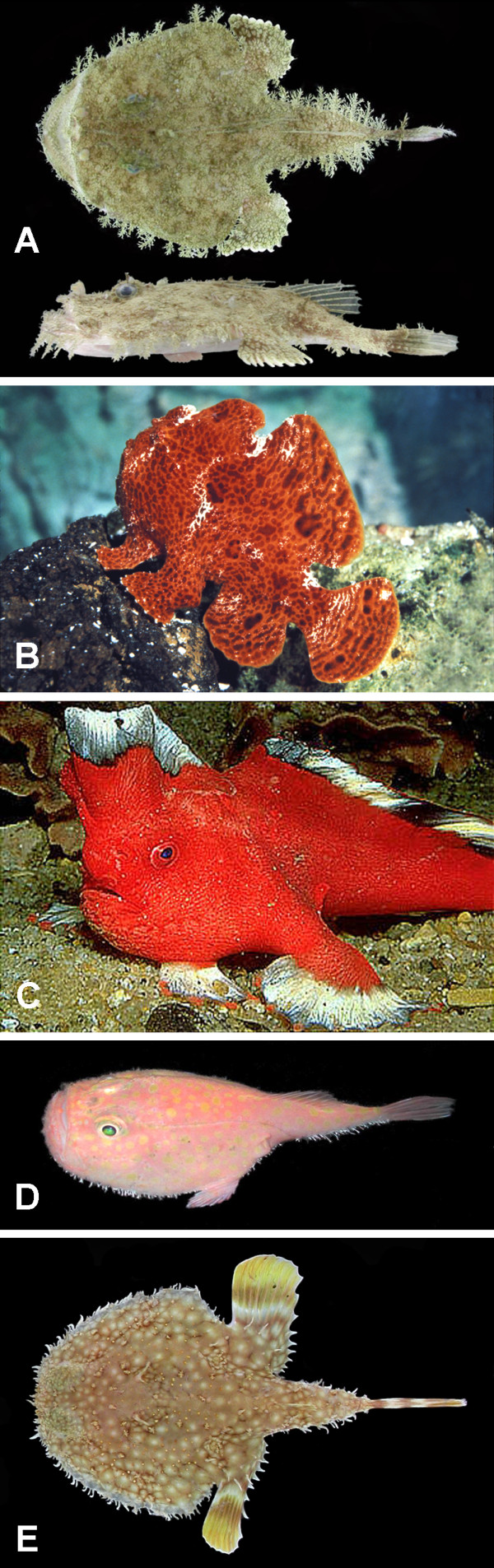
**Representatives of the lophiiform suborders Lophioidei (A), Antennarioidei (B, C), Chaunacoidei (D), and Ogcocephaloidei (E)**. (A) *Lophiodes reticulatus *Caruso and Suttkus, 157 mm SL, UF 158902, dorsal and lateral views (photo by J. H. Caruso); (B) *Antennarius commerson *(Latreille), 111 mm SL, UW 20983 (photo by D. B. Grobecker); (C) *Sympterichthys politus *(Richardson), specimen not retained (photo by R. Kuiter); (D) *Chaunax suttkusi *Caruso, 107 mm SL, TU 198058 (photo by J. H. Caruso); (E) *Halieutichthys aculeatus *(Mitchill), 80 mm SL, specimen not retained, dorsal view (photo by J. H. Caruso). Courtesy of the American Society of Ichthyologists and Herpetologists.

**Figure 2:**
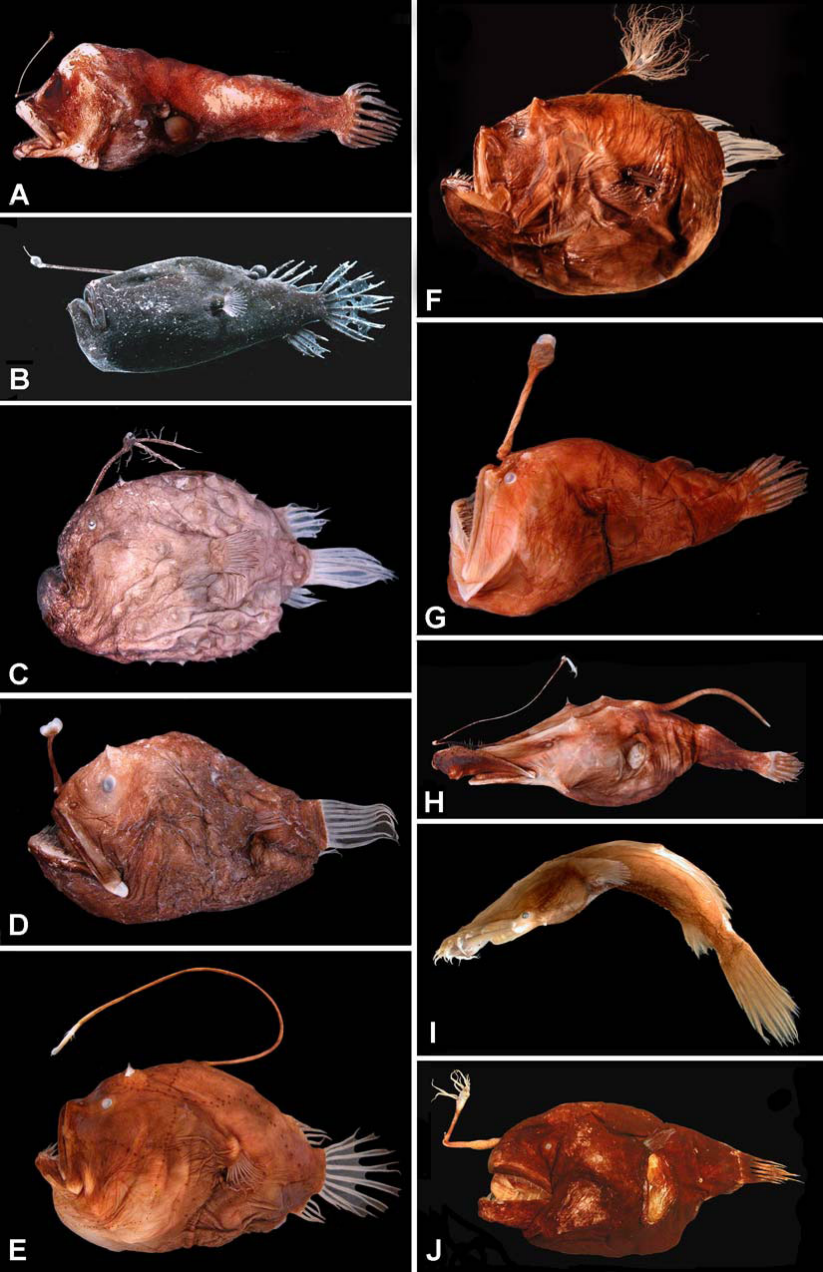
**Representatives of ceratioid families as recognized in this study-1**. (A) Centrophrynidae: *Centrophryne spinulosa *Regan and Trewavas, 136 mm SL, LACM 30379-1; (B) Ceratiidae: *Cryptopsaras couesii *Gill, 34.5 mm SL, BMNH 2006.10.19.1 (photo by E. A. Widder); (C) Himantolophidae: *Himantolophus appelii *(Clarke), 124 mm SL, CSIRO H.5652-01; (D) Diceratiidae: *Diceratias trilobus *Balushkin and Fedorov, 86 mm SL, AMS I.31144-004; (E) Diceratiidae: *Bufoceratias wedli *(Pietschmann), 96 mm SL, CSIRO H.2285-02; (F) Diceratiidae: *Bufoceratias shaoi *Pietsch, Ho, and Chen, 101 mm SL, ASIZP 61796 (photo by H.-C. Ho); (G) Melanocetidae: *Melanocetus eustales *Pietsch and Van Duzer, 93 mm SL, SIO 55-229; (H) Thaumatichthyidae: *Lasiognathus amphirhamphus *Pietsch, 157 mm SL, BMNH 2003.11.16.12; (I) Thaumatichthyidae: *Thaumatichthys binghami *Parr, 83 mm SL, UW 47537 (photo by C. Kenaley); (J) Oneirodidae: *Chaenophryne quasiramifera *Pietsch, 157 mm SL, SIO 72-180. Courtesy of the American Society of Ichthyologists and Herpetologists.

**Figure 3:**
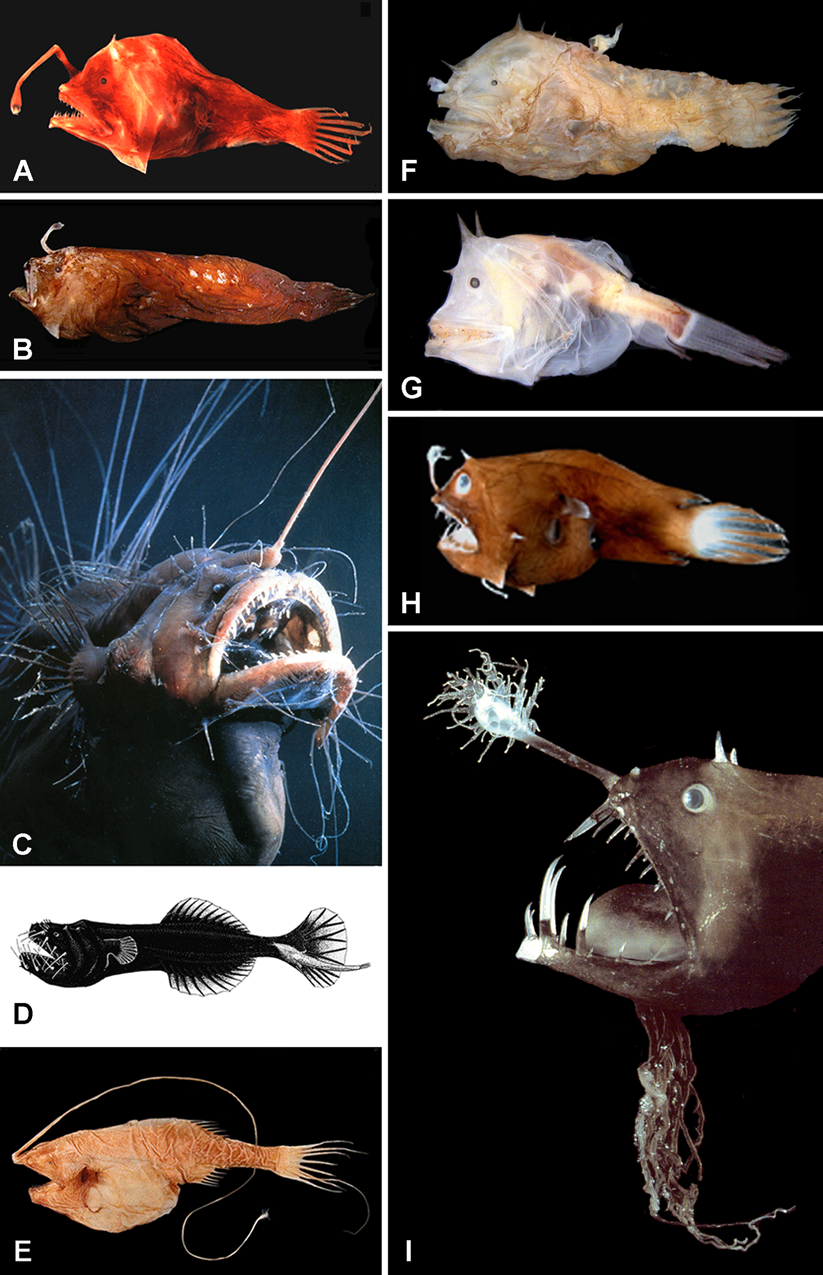
**Representatives of ceratioid families as recognized in this study-2**. (A) Oneirodidae: *Oneirodes *sp., 31 mm SL, MCZ 57783 (photo by C. P. Kenaley); (B) Oneirodidae: *Spiniphryne duhameli *Pietsch and Baldwin, 117 mm SL, SIO 60-239; (C) Caulophrynidae: *Caulophryne pelagica *(Brauer), 183 mm SL, BMNH 2000.1.14.106 (photo by D. Shale); (D) Neoceratiidae: *Neoceratias spinifer *Pappenheim, 52 mm SL, with 15.5-mm SL parasitic male, ZMUC P921726 (after Bertelsen, 1951); (E) Gigantactinidae: *Gigantactis gargantua *Bertelsen, Pietsch, and Lavenberg, 166 mm SL, LACM 9748-028; (F) Linophrynidae: *Photocorynus spiniceps *Regan, 46-mm SL, with 6.2-mm SL parasitic male, SIO 70-326; (G) Linophrynidae: *Haplophryne mollis *(Brauer), 36 mm SL, MNHN 2004-0811; (H) Linophrynidae: *Linophryne macrodon *Regan, 28 mm SL, UW 47538 (photo by C. P. Kenaley); (I) Linophrynidae: *Linophryne polypogon *Regan, 33 mm SL, BMNH 2004.9.12.167 (photo by P. David). Courtesy of the American Society of Ichthyologists and Herpetologists.

The order Lophiiformes contains a diverse array of marine fishes, ranging from benthic shallow-water dwellers to several groups of deep-shelf and slope inhabitants as well as a highly modified assemblage of open-water, meso- and bathypelagic species. Commonly referred to as anglerfishes, the group is characterized most strikingly by the structure of the first dorsal-fin spine, typically placed out on the tip of the snout and modified to serve as a luring apparatus for the attraction of prey. The order comprises approximately 325 living species, distributed among 68 genera and 18 families (Table 1). The families themselves are distributed among five suborders [1-3]: the Lophioidei (one family), relatively shallow-water, dorso-ventrally flattened forms, commonly referred to as the goosefishes or monkfishes (Figure 1A); the Antennarioidei (four families), nearly all laterally compressed, shallow- to moderately deep-water, benthic forms, with a host of common names including frogfishes (Figure 1B), sea-mice, sea-toads, warty anglerfishes, and handfishes (Figure 1C); the Chaunacoidei or coffinfishes (one family), more or less globose, deep-water benthic forms (Figure 1D); the Ogcocephaloidei or batfishes (one family), dorsoventrally flattened, deep-water benthic forms (Figure 1E); and the Ceratioidei (11 families), the deep-sea anglerfishes (Figures 2, 3), characterized most distinctly by their extremely dwarfed males attaching themselves (either temporarily or permanently) to the bodies of relatively gigantic females [4].

**Table 1:** Diversity of the Lophiiformes

Suborder	Family	Genus	%	Species	%
Lophioidei	Lophiidae	4 (4)	100.0	4 (25)	16.0
Antennarioidei	Antennariidae	2 (12)	16.7	3 (45)	6.7
	Tetrabrachiidae	1 (2)	50.0	1 (2)	50.0
	Brachionichthyidae	1 (2)	50.0	1 (5)	20.0
	Lophichthyidae	0 (1)	0.0	0 (1)	0.0
Chaunacoidei	Chaunacidae	1 (2)	50.0	3 (14)	21.4
Ogcocephaloidei	Ogcocephalidae	4 (10)	40.0	4 (68)	5.9
Ceratioidei	Caulophrynidae	1 (2)	50.0	2 (5)	40.0
	Neoceratiidae	1 (1)	100.0	1 (1)	100.0
	Melanocetidae	1 (1)	100.0	2 (6)	33.3
	Himantolophidae	1 (1)	100.0	2 (18)	11.1
	Diceratiidae	2 (2)	100.0	2 (6)	33.3
	Oneirodidae	4 (16)	25.0	4 (63)	6.3
	Thaumatichthyidae	2 (2)	100.0	2 (8)	25.0
	Centrophrynidae	1 (1)	100.0	1 (1)	100.0
	Ceratiidae	2 (2)	100.0	2 (4)	50.0
	Gigantactinidae	2 (2)	100.0	2 (21)	9.5
	Linophrynidae	3 (5)	60.0	3 (27)	11.1

	Total	33 (68)	48.5	39 (321)	12.1

Numbers of genera and species of 18 lophiiform families used in this study, with taxonomic diversity (numbers in parentheses) estimated by Pietsch [2]

Within the higher teleosts, the Lophiiformes has traditionally been allied with toadfishes of the order Batrachoidiformes, based primarily on osteological characters of the cranium [5-7]. Following the publication of the seminal work on higher-level relationships of teleosts by Greenwood et al. [8] and the advent of cladistic theory [9], both groups have been placed in the Paracanthopterygii, a presumed sister-group of the more derived Acanthopterygii [7]. Other than the Lophiiformes and Batrachoidiformes, the original Paracanthopterygii [7] included those groups of fishes thought to be relatively primitive in the higher teleosts, such as Polymixiiformes, Percopsiformes, Ophidiiformes, Gadiformes, Zeioidei, Zoarcoidei and Gobiesocoidei. Subsequently, the taxonomic contents of the Paracanthopterygii have undergone significant changes, being finally reduced to five core orders (Percopsiformes, Ophidiiformes, Gadiformes, Batrachoidiformes, Lophiiformes) in an attempt to make the group monophyletic [10], and this taxonomic proposal has been followed in many reference books [11-14]. Thus the paracanthopterygian Lophiiformes (and its close association with the Batrachoidiformes) has been a prevailing view in the ichthyological community despite the lack of convincing evidence [1,15,16].

Recent molecular phylogenetic studies, however, have repeatedly cast doubt on such a paracanthoperygian position of the Lophiiformes within the higher teleosts [17-27]. These studies based on nucleotide sequences from both whole mitogenomes and various nuclear genes have strongly suggested that lophiiforms are highly derived teleosts, deeply nested in one of the larger percomorph clades, and that they are closely related to various percomorphs, such as the Tetraodontiformes, Caproidei, Acanthuroidei, Chaetodontidae, Pomacanthidae, Ephippidae and Drepanidae, all of them showing no indications of close affinity with the Lophiiformes before the advent of molecular phylogenetics. Significantly a mitochondrial phylogenomic study by Miya et al. [25] demonstrated that the Batrachoidiformes was deeply nested within a different percomorph clade consisting of the Synbranchiformes and Indostomiidae and a sister-group relationship between the Lophiiformes and Batrachoidiformes was confidently rejected by the Bayesian analyses. These novel relationships, however, have not been reflected in the most recently published classification of fishes [14].

**Figure 4:**
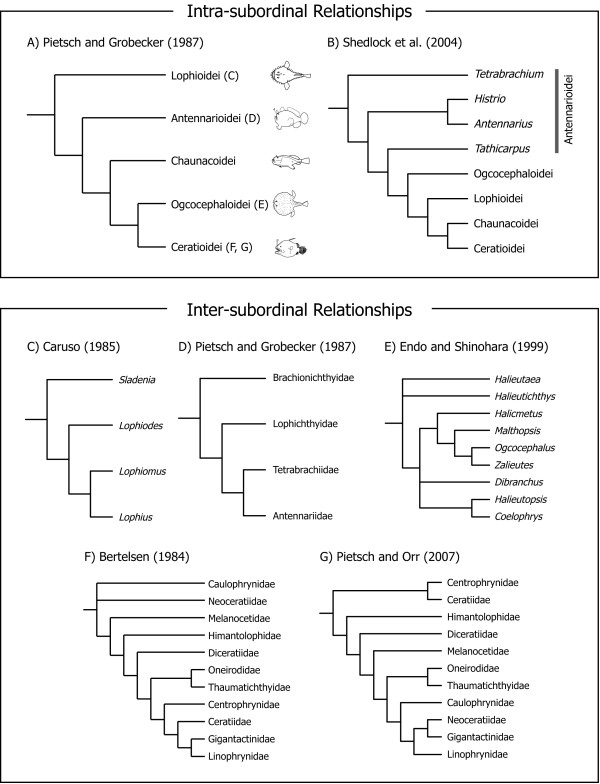
**Previously proposed phylogenetic hypotheses within the Lophiiformes**. Inter-subordinal relationships based on (A) morphology [3] and (B) the mitochondrial 16 rDNA sequences [29]. Intra-subordinal relationships based on (C) morphologies for the Lophioidei [30], (D) Antennarioidei [3], (E) Ogcocephaloidei [31] and (F, G) Ceratioidei [32,33].

Within the Lophiiformes, interrelationships among 18 families and five suborders have been inadequately studied, owing to limited availability of specimens from the most taxonomically rich suborder Ceratioidei. Nevertheless Pietsch and his colleagues [1,3,28] have analyzed morphological characters in several attempts to resolve subordinal and family relationships. In their preferred cladogram, the Lophioidei occupies the most basal position, followed by Antennarioidei and Chaunacoidei, with the Ogcocephaloidei and Ceratioidei forming a sister-group at the top of the tree (Figure 4A). More recently Shedlock et al. [29] compared short fragments of the mitochondrial 16S rRNA genes from 18 lophiiforms including all five suborders, and analyzed 513 aligned nucleotide sites using the maximum likelihood (ML) method, with two batrachoidiforms species as outgroups. The resulting tree (Figure 4B), however, significantly departed from both the results based on morphological (Figure 4A) and molecular data [24-26], although the latter studies dealt with only six species in three suborders (Lophioidei, Chaunacoidei, Ceratioidei). Within each subordinal lineage, several authors have published phylogenetic hypotheses based on morphological characters (Figure 4C-G), including those of Caruso [30] for the Lophioidei, Pietsch and Grobecker [3] for the Antennarioidei, Endo and Shinohara [31] for the Ogcocephaloidei, Bertelsen [32] and Pietsch and Orr [33], and Pietsch [2] for the Ceratioidei. There has been no attempt, however, to resolve their phylogenies using molecular data.

In addition to the lack of available material of numerous rare taxa, the evolutionary history of the lophiiform fishes has remained elusive because of poor representation in the fossil record (but see [34-38]). Recent developments in the molecular estimation of divergence times, however, have provided promising tools to introduce time scales for the phylogenetic trees [39], thereby offering new insights into evolutionary history that cannot be inferred by the fossil data alone. Among the most significant advances common to these new methods is a departure from the molecular clock assumption and the use of time constraints at multiple nodes for rate calibration, usually based on fossil record. In higher teleosts, however, including lophiiforms, the fossil record is scarce and fragmentary, and alternative calibration points based on biogeographic events have proven useful for divergence time estimation. Azuma et al. [40] recently found that estimated divergence times of cichlid fishes showed excellent agreement with the history of Gondwanian fragmentation, arguing that such biogeographic events can be used as effective time constraints in dating teleostean divergences, which may be useful for dating lophiiform divergence times.

To address questions regarding the subordinal and familial relationships and evolutionary history of the Lophiiformes, we assembled the whole mitochondrial genome sequences from the 39 lophiiform species (33 sequences newly-determined during this study), representing all of the five suborders and 17 of the 18 families. Unambiguously aligned sequences (14,611 bp) from those 39 species plus 38 outgroups (total 77 species) were subjected to partitioned maximum likelihood (ML) analysis using RAxML [41]. The resulting tree topology was then used to estimate the divergence time of the Lophiiformes using a Bayesian relaxed molecular-clock method to infer their evolutionary history, and patterns and rates of diversifications.

## Methods

### Taxon sampling

Our taxon sampling followed results from recent mitochondrial phylogenomic studies by Miya et al. [25,26] who first proposed that the Lophiiformes was a highly advanced percomorph group and confidently rejected their affinity with paracanthopterygians. They also proposed that the order was closely related to members of previously unallied groups such as Caproidei and Tetraodontiformes, a hypothesis that was subsequently supported by Yamanoue et al. [27] in their study of Tetraodontiformes based on the 44 whole mitogenome sequences. Thus, in the present study, we incorporated all of the 44 species (including six lophiiforms) used by Yamanoue et al. [27] and added 33 species of lophiiforms for a total 77 species (Table 2). Despite limited availability of fresh materials from bathypelagic ceratioids, we were able to collect tissues of all 11 families, lacking for the entire order only the rare monotypic antennarioid family Lophichthyidae (Table 1). Accordingly, we sampled all of the five suborders, 17 of the 18 families (94.4%), 33 of the 68 genera (48.5%), and 39 of the 321 species (12.1%), a coverage sufficient to address higher-level relationships of the Lophiiformes. We acknowledge that the taxon sampling is still sparse for three species-rich families, the Antennariidae (6.7%), Ogcocephalidae (5.9%), and Oneirodidae (6.3%) (see Table 1). The final rooting was done using a non-percomorph *Polymixia japonica *(Polymixiidae).

**Table 2:** List of species used in this study

Family ^a^	Species	Accession No.
**Outgroup (38 spp.)**		
Order Polymixiiformes		
Polymixiidae	*Polymixia japonica*	AB034826
Order Beryciformes		
Berycidae	*Beryx splendens*	AP002939
Order Scorpaeniformes		
Triglidae	*Satyrichthys amiscus*	AP004441
Order Perciformes		
Suborder Zoarcoidei		
Zoarcidae	*Enedrias crassispina*	AP004449
Suborder Percoidei		
Centropomidae	*Coreoperca kawamebari*	AP005990
Acropomatidae	*Doederleinia berycoides*	AP009181
Lutjanidae	*Lutjanus rivulatus*	AP006000
	*Pterocaesio tile*	AP004447
Emmelichthyidae	*Emmelichthys struhsakeri*	AP004446
Haemulidae	*Diagramma pictum*	AP009167
	*Parapristipoma trilineatum*	AP009168
Sparidae	*Pagrus major*	AP002949
Centracanthidae	*Spicara maena*	AP009164
Lethrinidae	*Lethrinus obsoletus*	AP009165
	*Monotaxis grandoculis*	AP009166
Monodactylidae	*Monodactylus argenteus*	AP009169
Chaetodontidae	*Chaetodon auripes*	AP006004
	*Heniochus diphreutes*	AP006005
Pomacanthidae	*Chaetodontoplus septentrionalis*	AP006007
	*Centropyge loriculus*	AP006006
Suborder Acanthuroidei		
Luvaridae	*Luvarus imperialis*	AP009161
Zanclidae	*Zanclus cornutus*	AP009162
Acanthuridae	*Naso lopezi*	AP009163
	*Zebrasoma flavescens*	AP006032
Suborder Caproidei		
Caproidae	*Antigonia capros*	AP002943
	*Capros aper*	AP009159
Order Tetraodontiformes		
Superfamily Triacanthoidea		
Triacanthodidae	*Triacanthodes anomalus*	AP009172
	*Macrorhamphosodes uradoi*	AP009171
Triacanthidae	*Triacanthus biaculeatus*	AP009174
	*Trixiphichthys weberi*	AP009173
Superfamily Balistoidea		
Balistidae	*Sufflamen fraenatum*	AP004456
Monacanthidae	*Stephanolepis cirrhifer*	AP002952
Ostraciidae	*Ostracion immaculatus*	AP009176
	*Kentrocapros aculeatus*	AP009175
Superfamily Triodontidae		
Triodontidae	*Triodon macropterus*	AP009170
Tetraodontidae	*Takifugu rubripes*	AP006045
Diodontidae	*Diodon holocanthus*	AP009177
Molidae	*Ranzania laevis*	AP006047
**Ingroup (39 spp.)**		
Order Lophiiformes		
Suborder Lophioidei		
Lophiidae	*Lophius americanus*	AP004414
	*Lophiomus setigerus *^b^	AP004413
	*Lophiodes caulinaris*	AB282826
	*Sladenia gardineri*	AB282827
Suborder Antennarioidei		
Antennariidae	*Antennarius striatus*	AB282828
	Antennarius coccineus*	AB282830
	*Histrio histrio*	AB282829
Tetrabrachiidae	*Tetrabrachium ocellatum*	AB282831
Brachionichthyidae	Brachionichthys hirsutus*	AB282832
Suborder Chaunacoidei		
Chaunacidae	*Chaunax abei*	AP004415
	Chaunax tosaensis*	AP004416
	Chaunax pictus*	AB282833
Suborder Ogcocephaloidei		
Ogcocephalidae	*Malthopsis jordani*	AP005978
	Halieutaea stellata*	AP005977
	Coelophrys brevicaudata*	AB282834
	*Zalieutes elater*	AB282835
Suborder Ceratioidei		
Caulophrynidae	*Caulophryne jordani *^c^	AP004417
	Caulophryne pelagica*	AB282836
Neoceratiidae	Neoceratias spinifer*	AB282837
Melanocetidae	*Melanocetus murrayi*	AP004418
	*Melanocetus johnsonii*	AB282838
Himantolophidae	*Himantolophus albinares*	AB282839
	*Himantolophus groenlandicus*	AB282840
Diceratiidae	Bufoceratias thele*	AB282841
	*Diceratias pileatus*	AB282842
Oneirodidae	*Oneirodes thompsoni*	AB282843
	*Puck pinnata*	AB282844
	*Chaenophryne melanorhabdus*	AB282845
	*Bertella idiomorpha*	AB282846
Thaumatichthyidae	*Thaumatichthys pagidostomus*	AB282847
	*Lasiognathus *sp.	AB282848
Centrophrynidae	*Centrophryne spinulosus*	AB282849
Ceratiidae	*Cryptopsaras couesii*	AB282850
	*Ceratias uranoscopus*	AB282851
Gigantactinidae	*Gigantactis vanhoeffeni*	AB282852
	*Rhynchactis macrothrix*	AB282853
Linophrynidae	*Linophryne bicornis*	AB282854
	*Acentrophryne dolichonema*	AB282855
	*Haplophryne mollis*	AB282856

^a ^Classification follows Nelson [14] except for recognition of five suborders in the Lophiiformes [2].

^b ^Originally published as *Lophius litulon *by Miya et al. [26], but subsequently reidentified as *Lophiomus setigerus *by MM based on reexamination of the voucher specimen (CBM-ZF 10732).

^c ^Originally published as *Caulophryne pelagica *by Miya et al. [26], but subsequently reidentified as *C. jordani *by TWP based on reexamination of the voucher specimen (CBM-ZF 12209).

* Those species used for divergence time estimation for crown nodes of the Lophiiformes and its five suborders.

### DNA extraction, PCR, and Sequencing

We excised a small piece of epaxial musculature or fin-ray (ca. 0.25 g) from fresh or ethanol-fixed specimens of each species and preserved them in 99.5% ethanol. We extracted total genomic DNA from the tissue using QIAamp or DNeasy (Qiagen) following the manufacturer's protocol. We amplified the mitogenomes of the 33 lophiiform species in their entirety using a long PCR technique [42]. We basically used seven fish-versatile PCR primers for the long PCR in the following four combinations (for locations of these primers, see [43-46]): L2508-16S (5'-CTC GGC AAA CAT AAG CCT CGC CTG TTT ACC AAA AAC-3') + H12293-Leu (5'-TTG CAC CAA GAG TTT TTG GTT CCT AAG ACC-3'); L2508-16S + H15149-CYB (5'-GGT GGC KCC TCA GAA GGA CAT TTG KCC TCA-3'); L8343-Lys (5'-AGC GTT GGC CTT TTA AGC TAA WGA TWG GTG-3') + H1065-12S (5'-GGC ATA GTG GGG TAT CTA ATC CCA GTT TGT-3'); and L12321-Leu (5'-GGT CTT AGG AAC CAA AAA CTC TTG GTG CAA-3') + S-LA-16S-H (5'-TGC ACC ATT RGG ATG TCC TGA TCC AAC ATC-3'). When we failed to cover the entire mitogenomes with these primer pairs, we used an additional five long PCR primers specifically designed to amplify the lophiiform mitogenomes: H8319-ANG-Lys (5'-GKA GKC ACC AKT TTT TAG MTT AAA AGG C-3'); L7567-ANG-Asp (5'-ACG CTG TTK TGT CAA GGC ARR AYT GTG GGT-3'); L10054-ANG-Gly (5'-CAC CWG GTC TTG GTT WAA MTC CMA GGA AAG-3'); H15149-ANG-CYB (5'-AGG TTK GTG ATG ACK GTK GCK CCT CA-3'); and L14850-ANG-CYB (5'-AAT ATC TCG GTK TGG TGG AAY TTT GGK TC-3'). Long PCR reaction conditions followed Miya and Nishida [47]. Dilution of the long PCR products with TE buffer (1:10 to 100 depending on the concentration of the long PCR products) served as templates for subsequent short PCRs.

We used a standard set of 24 pairs of fish-versatile primers for short PCRs to amplify contiguous, overlapping segments of the entire mitogenome for each lophiiform species (Table 3). When some of the short PCR reaction failed, we managed to amplify those regions with the existing fish-versatile primers. We designed new species-specific primers when none of the primer pairs amplified the short segments. Short PCR reaction conditions followed Miya and Nishida [47]. A list of PCR primers for each species is available upon request to MM.

**Table 3:** Standard set of 24 short PCR primer pairs for lophiiforms

No.	Primer ^a^	Sequence (5' to 3')	Reference
1	L1083-12S	ACAAACTGGGATTAGATAC	[47]
	H2590-16S	ACAAGTGATTGCGCTACCTT	[47]
2	L2949-16S	GGGATAACAGCGCAATC	[47]
	H3976-ND1	ATGTTGGCGTATTCKGCKAGGAA	[43]
3	L2949-16S	GGGATAACAGCGCAATC	[47]
	H4432-Met	TTTAACCGWCATGTTCGGGGTATG	[46]
4	L4299-Ile	AAGGRCCACTTTGATAGAGT	This study
	H5669-Asn	AACTGAGAGTTTGWAGGATCGAGGCC	[53]
5	L4633-ND2	CACCACCCWCGAGCAGTTGA	[47]
	H5669-Asn	AACTGAGAGTTTGWAGGATCGAGGCC	[53]
6	L5549-Trp	AAGACCAGGAGCCTTCAAAG	This study
	H6558-CO1	CCKCCWGCKGGGTCAAAGAA	[53]
7	L6205-CO1	TTCCCWCGAATAAATAACATAAG	[87]
	H7447-Ser	AWGGGGGTTCRATTCCTYCCTTTCTC	[87]
8	L7255-CO1	GATGCCTACACMCTGTGAAA	[47]
	H8312-Lys	CACCWGTTTTTGGCTTAAAAGGCTAAYGCT	[87]
9	L8202-CO2	TGYGGAGCWAATCAYAGCTT	[87]
	H9375-CO3	CGGATRATGTCTCGTCATCA	[53]
10	L8343-Lys	AGCGTTGGCCTTTTAAGCTAAWGATWGGTG	[87]
	H9639-CO3	CTGTGGTGAGCYCAKGT	[47]
11	L8343-Lys	AGCGTTGGCCTTTTAAGCTAAWGATWGGTG	[87]
	H10019-Gly	CAAGACKGKGTGATTGGAAG	[47]
12	L8343-Lys	AGCGTTGGCCTTTTAAGCTAAWGATWGGTG	[87]
	H10433-Arg	AACCATGGWTTTTTGAGCCGAAAT	[47]
13	L10054-Gly	CACCWGGTCTTGGTTWAAMTCCMAGGAAAG	This study
	H11534-ND4M	GCTAGKGTAATAAWKGGGTA	[87]
14	L10440-Arg	AAGATTWTTGATTTCGGCT	[27]
	H11534-ND4M	GCTAGKGTAATAAWKGGGTA	[87]
15	L11424-ND4	TGACTTCCWAAAGCCCATGTAGA	[47]
	H12632-ND5	GATCAGGTTACGTAKAGKGC	[47]
16	L12329-Leu	CTCTTGGTGCAAMTCCAAGT	[47]
	H13396-ND5	CCTATTTTTCGGATGTCTTG	[53]
17	L12329-Leu	CTCTTGGTGCAAMTCCAAGT	[47]
	H13727-ND5	GCGATKATGCTTCCTCAGGC	[47]
18	L13553-ND5	AACACMTCTTAYCTWAACGC	[53]
	H14768-CYB	TTKGCGATTTTWAGKAGGGGGTG	[87]
19	L13553-ND5	AACACMTCTTAYCTWAACGC	[53]
	H15149-CYB	GGTGGCKCCTCAGAAGGACATTTGKCCTCA	[53]
20	L14504-ND6	GCCAAWGCTGCWGAATAMGCAAA	[53]
	H15560-CYB	TAGGCRAATAGGAARTATCA	[47]
21	L14718-Glu	TTTTTGTAGTTGAATWACAACGGT	This study
	H15913-Thr	CCGGTSTTCGGMTTACAAGACCG	[87]
22	L15369-CYB	ACAGGMTCAAAYAACCC	[53]
	H16484-CR	GAGCCAAATGCMAGGAATARWTCA	[87]
23	L15998-Pro	AACTCTTACCMTTGGCTCCCAARGC	[53]
	H885-12S	TAACCGCGGYGGCTGGCACGA	[87]
24	L16507-CR	TGAWYTATTCCTGGCATTTGGYTC	[87]
	H1358-12S	CGACGGCGGTATATAGGC	[47]

^a ^L and H denote light and heavy strands, respectively. Positions with mixed bases are labeled with their IUB codes

We purified double-stranded short PCR products using a Pre-Sequencing kit (USB) for direct cycle sequencing with dye-labeled terminators (Applied Biosystems). We performed all sequencing reactions according to the manufacture's instructions with the same primers as those for the short PCRs. We analyzed labeled fragments on model 373/377/3100/3130*xl *sequencers (Applied Biosystems).

### Sequence editing and alignment

We edited each sequence electropherogram with EditView (ver. 1.01; Applied Biosystems) and concatenated the multiple sequences using AutoAssembler (ver. 2.1; Applied Biosystems). We carefully checked the concatenated sequences using DNASIS (ver. 3.2; Hitachi Software Engineering) and created a sequence file for each gene. We compared the sequence files among closely related species to minimize sequence errors. Genes (or a portion of genes) that we were unable to sequence owing to technical difficulties were coded as missing.

To check sensitivity of additional taxon sampling of a number of the lophiiforms to the results reported in Yamanoue et al. [27], we used their pre-aligned sequences as a basis for further alignment with the newly determined sequences from 33 lophiiforms. Yamanoue et al. [27] aligned 13 protein-coding, two rRNA, and 22 tRNA genes using ProAlign ver. 0.5 [48] and they used only those positions with posterior probabilities ≥70%. An exception to this was the alignment of tRNA genes, for which Yamanoue et al. [27] modified the alignment on the basis of the secondary structure, estimated with DNASIS. They used all the stem regions even if the aligned sequences were <70% posterior probabilities. Because the aligned sequences of Yamanoue et al. [27] included several overlapping positions between the two open reading frames (ATPase 8/6, ND4L/4, and ND5/6), we excluded those positions from the downstream genes (ATPase 6, ND4, and ND6).

To combine pre-aligned sequences from Yamanoue et al. [27] with the new sequences, we rearranged the dataset of Yamanoue et al. [27] into typical gene order of vertebrates (beginning from tRNA-Phe) and saved it in a FASTA format. The 33 newly determined sequences in the same format were concatenated to the rearranged, pre-aligned sequences and the dataset was subjected to the multiple alignment using MAFFT ver. 6 [49]. We imported the aligned sequences into MacClade ver. 4.08 [50] and removed the redundant regions appeared as gaps with slight modifications by eye to correctly reproduce the aligned sequences used in Yamanoue et al. [27]. All the resulting gap positions from the alignment were coded as missing.

### Phylogenetic analysis

We divided unambiguously aligned sequences into five partitions (first, second, third codon positions, rRNA and tRNA genes) assuming that functional constraints on sequence evolution are more similar within codon positions (or types of molecules) across genes than across codon positions (or types of molecules) within genes. We converted nucleotides at the third codon positions into purine (A/G) with "R" and pyrimidine (C/T) with "Y" (RY-coding; Phillips and Penny [51]) to take only transversions into account in the phylogenetic analysis following the recommendation of Saitoh et al. [52]. This coding effectively removes likely "noise" from the dataset [53], and avoids the apparent lack of signal by retaining all available positions in the dataset. The "R" and "Y" were further recoded with "A" and "C," respectively, to avoid unnecessary estimation of transitional changes during the calculations using RAxML with the exception of an outgroup species (*Polymixia japonicus*) for running the program (RAxML does not accept all "A/C" partitions). We also constructed an additional two datasets that treat quickly saturated third codon positions differently (with or without third codon positions) to check sensitivity of the datasets to the phylogenetic analysis. The three datasets are designated as follows: 1) RY-coding (12_n_3_r_RT_n_); 2) all positions included (123_n_RT_n_); and 3) third codon positions excluded (12_n_RT_n_).

We subjected the above datasets to the partitioned maximum-likelihood (ML) analysis using RAxML ver. 7.0.4 [41]. A general time reversible model with sites following a discrete gamma distribution (GTR + Γ; the model recommended by the author) was used and a rapid bootstrap (BS) analysis was conducted with 500 replications (-f a option). This option performs BS analysis using GTRCAT, which is GTR approximation with optimization of individual per-site substitution rates, and classification of those individual rates into certain number of rate categories. After implementing the BS analysis, the program uses every fifth BS tree as a starting point to search for the ML tree using GTR + Γ model of sequence evolution and saves the top 10 best-scoring ML trees (fast ML searches). Finally RAxML calculates more correct likelihood scores (slow ML searches) for those 10 trees and puts BS probabilities on the best-scoring ML tree.

### Testing alternative phylogenetic hypotheses

We considered that the best-scoring ML tree resulting from 12_n_3_r_RT_n _(RY-coding) dataset as the best estimate of phylogeny because this coding effectively removes likely "noise" from the dataset and avoids the apparent lack of signal by retaining all available positions in the dataset (see discussions in Saitoh et al. [52]). Alternative tree topologies were thus individually compared to the resulting best-scoring ML tree derived from the 12_n_3_r_RT_n _dataset using the likelihood-based approximately unbiased (AU) test as implemented in CONSEL [54]. The *p-*values from this test are calculated using the multi-scale bootstrap technique and are less biased than those of the conventional methods such as the bootstrap probability (BP), the Kishino-Hasegawa (KH) test and the Shimodaira-Hasegawa (SH) test [55].

We manually created the constrained tree topologies with reference to the alternative hypotheses using MacClade and then performed the RAxML analysis with each constraint using the -g option. We conducted the fast bootstrappings with 100 replicates as described above and the resulting best-scoring ML tree was considered as the constrained ML tree. The constrained and unconstrained ML trees (best-scoring ML tree without constraint) were used to compute the per-site log likelihood scores for each tree using the -f g option in RAxML and the output was subjected to CONSEL analysis to calculate statistical significance of the differences in the likelihood scores.

### Tracing character evolution

Male sexual parasitism has been found among only the Ceratioidei [4]. Its evolution was reconstructed on the best-scoring ML tree derived from 12_n_3_r_RT_n _dataset under an ML optimality criterion using Mesquite ver. 2.6 [56]. The ML reconstruction methods find the ancestral states that maximize the probability the observed states would evolve under a stochastic model of evolution [57,58]. The Mk1 model ("Markov k-state 1 parameter model"), a k-state generalization of the Jukes-Cantor model that corresponds to Lewis's Mk model [59], was used to trace the character evolution. Four character states were assigned to the male sexual parasitism based on extensive observations made by Pietsch [4] and Pietsch and Orr [33]: males never attach to females (character state 0); males attach temporarily (state 1); males are facultative parasites (state 2); and males are obligate parasites (state 3).

### Divergence time estimation

Because lophiiforms are rarely represented in the fossil record [34-37], the age of divergence of the lophiiform clades cannot be established precisely based on paleontological data alone. Thus external calibration points should be used at multiple nodes to estimate the divergence times of the Lophiiformes correctly. To that end, we used the mitogenomic dataset of Azuma et al. [40] who extensively sampled actinopterygians from the base to the top of the tree. Significantly the dataset of Azuma et al. [40] includes 1) all major lineages of the basal actinopterygians whose fossils and their relative phylogenetic positions are more reliable than those of the higher teleosts; and 2) all continental cichlids whose divergences show excellent agreement with the history of Gondwanian fragmentations.

Mitogenome sequences from the 39 lophiiforms were concatenated with the pre-aligned sequences used in Azuma et al. [40] in a FASTA format and the dataset was subjected to multiple alignment using MAFFT ver. 6 [49] as described above. The dataset comprises 6966 positions from first and second codon positions of the 12 protein-coding genes, 1673 positions from the two rRNA genes and 1407 positions from the 22 tRNA genes (total 10,046 positions). The third codon positions of the protein-coding genes were entirely excluded because of their extremely accelerated rates of changes that may cause a high level of homoplasy at this taxonomic scale [53] and overestimation of divergence times [60].

Ideally all node ages for the 39 lophiiform species can be estimated in a single step; however, recent studies demonstrated that dense taxon sampling in a particular lineage (as has been done for the Lophiiformes in this study) tend to lead to overestimation of its age compared to the rest of the tree ("node-density effect" [61,62]). To avoid such unnecessary overestimation, we retained a minimum number of taxa from each suborder in proportion to the logarithms of the species' diversity (Table 1). We selected the most distantly related species from each suborder to estimate crown node ages as correctly as possible. The nine selected species (three species from the most species-rich Ceratioidei and two from the rest of four suborders) are shown in Table 2 with asterisks. The resulting dataset contains 54 species used in Azuma et al [40] plus nine lophiiforms, with the total number of species being 63.

We used a relaxed molecular-clock method for dating analysis developed by Thorne and Kishino [63] to estimate divergence times. This method accommodates unlinked rate variation across different loci ("partitions" in this study), allows the use of time constraints on multiple divergences, and uses a Bayesian MCMC approach to approximate the posterior distribution of divergence times and rates based on a single tree topology estimated from the other method (ML tree in this study). A series of application in the software package multidistribute (v9/25/2003) were used for these analyses.

Baseml in PAML ver. 3.14 was used to estimate model parameters for each partition separately under the F84 + Γ model of sequence evolution (the most parameter-rich model implemented in multidistribute). Based on the outputs from baseml, branch lengths and the variance-covariance matrix were estimated using estbranches in multidistribute for each partition. Finally multidivtime in multidistribute was used to perform Bayesian MCMC analyses to approximate the posterior distribution of substitution rates, divergence times, and 95% credible intervals. In this step, multidivtime uses estimated branch lengths and the variance-covariance matrices from all partitions without information from the aligned sequences.

MCMC approximation with a burnin period of 100,000 cycles was obtained and every 100 cycles was taken to create a total of 10,000 samples. To diagnose possible failure of the Markov chains to converge to their stationary distribution, at least two replicate MCMC runs were performed with two different random seeds for each analysis.

Application of multidivtime requires values for the mean of the prior distribution for the time separating the ingroup root from the present (rttm) and its standard deviation (rttmsd), and we set conservative estimates of 4.2 (= 420 Myr ago [Ma]) and 4.2 SD, respectively. The tip-root branch lengths were calculated using TreeStat v. 1.1 http://tree.bio.ed.ac.uk/software/treestat/ for all terminals and their average was divided by rttm (4.2) to estimate rate of the root node (rtrate) and its standard deviation (rtratesd), which were set to 0.074 and 0.074, respectively. The priors for the mean of the Brownian motion constant, brownmean and brownsd, were both set to 0.5, specifying a relatively flexible prior.

The multidivtime program allows for both minimum (lower) and maximum (upper) time constraints and it has been argued that multiple calibration points would provide overall more realistic divergence time estimates. We therefore sought to obtain an optimal phylogenetic coverage of calibration points across our tree, although we could set maximum constraints based on fossil records only for the three basal splits between Sarcopterygii and Actinopterygii, Polypteriformes and Actinopteri, Acipenseriformes and Neopterygii (A-C in Table 4). We also set lower and upper time constraints for three nodes in cichlid divergence, which show excellent agreement with the Gondwanian fragmentation, assuming that they have never dispersed across oceans. Accordingly we set a total of 31 time constrains based on both the fossil record and biogeographic events as shown in Table 4. The resulting node ages for the Lophiiformes and its five suborders (posterior means) were used as the time constraints to estimate divergence times of all the 39 lophiiform species.

**Table 4:** List of time constraints used in divergence time estimation

Node	Constraints ^a^	Calibration information
A	U 472	The minimum age for the basal split of bony fish based on the earliest known acanthodian remains from Late Ordovician [88]
	L 419	The †*Psarolepis *fossil (sarcopterygian [89]) from Ludlow (Silurian) [90]
B	U 419	The minimum age for the Sarcopterygii/Actinopterygii split
	L 392	The †*Moythomasia *fossil (actinopteran) from the Givetian/Eifelian boundary [90]
C	U 392	The minimum age for the Polypteriformes/Actinopteri split
	L 345	The †*Cosmoptychius *fossil (neopterygian or actinopteran) from Tournasian [90]
D	L 130	The †*Protopsephurus *fossil (Polyodontidae) from Hauterivian (Cretaceous) [90]
E	L 284	The †*Brachydegma *fossil (stem amiids) from Artinskian (Permian) [90]
F	L 136	The †*Yanbiania *fossil (Hiodontidae) from the Lower Cretaceous [90]
G	L 112	The †*Laeliichthys *fossil (Osteoglossidae) from the Aptian (Cretaceous) [91]
H	L 151	The †*Anaethalion*, †*Elopsomolos*, and †*Eoprotelops *fossil (Elopomorpha) from Kimmeridgian (Jurassic) [90]
I	L 94	The †*Lebonichthys *(Albulidae) fossil from the Cenomanian (Cretaceous) [91]
J	L 49	The *Conger *(Congridae) and *Anguilla *(Anguillidae) fossils from the Ypresian (Tertiary) [91]
K	L 146	The †*Tischlingerichthys *fossil (Ostariophysi) from Tithonian (Jurassic) [90]
L	L 56	The †*Knightia *fossil (Clupeidae) from the Thanetian (Tertiary) [91]
M	L 49	The †*Parabarbus *fossil (Cyprinidae) from the Ypresian (Tertiary) [91]
N	L 74	The †*Esteseox foxi *fossil (Esociformes) from the Campanian (Cretaceous) [92]
O	L 94	The †*Berycopsis *fossil (Polymixiidae) from the Cenomanian (Cretaceous) [91]
P	L 50	The pleuronectiform fossil from the Ypresian (Tertiary) [91]
Q	L 98	The tetraodontiform fossil from the Cenomanian [83]
R	L 32	The estimated divergence time between *Takifugu *and *Tetraodon *[93]
S	U 95 L 85	The upper and lower bounds of separation between Madagascar and Indian [85,86,94]
T	U 145 L 112	The upper and lower bounds of separation between Indo-Madagascar landmass and Gondwanaland [85,86,94]
U	U 120 L 100	The upper and lower bounds of separation between African and South American landmasses [85,86]
V	L 40	The lophiid fossil from Lutetian (Eocene) [95]
W	L 40	The *Brachionichthys *fossil from Lutetian (Eocene) [28,34,95]
X	L 40	The ogcocephalid fossil from Lutetian (Eocene) [95]
Y	L 7.6	The ceratioid fossil from upper Mohnian [38]

^a ^U and L indicate maximum and minimum time constrains in million years (Myr), respectively (see Figure 9 for corresponding nodes).

### Net diversification rates

We estimated per-clade net diversification rates (*r *= *b *- *d*, where *b *is the speciation rate and *d *is the extinction rate) under relative extinction rates (*ε *= *d*/*b*) of 0 and 0.95 using Magallón and Sanderson's [64] method-of-moment estimator for each suborder. The equation is derived from

where *n *is the final number of lineages (present-day species diversity; Table 1) and *t *is the time interval considered (stem-group age).

## Results and discussion

In the following sections, we describe and discuss the mitogenomic phylogenies and evolutionary history of the Lophiiformes. Whole mitogenomic phylogenetic analysis has been extremely useful in illuminating new ideas of interrelationships of fishes in particular, and renewed morphological analysis of these proposed relationships has often provided additional morphological support to challenge prevailing ideas of evolutionary relationships [27,65]. We acknowledge, however, a phylogeny derived from the whole mitogenome only represents the mtDNA genealogy and may not necessarily match the evolution of the species under analysis. Because of the lack of recombination, the entire molecule of mtDNA has one molecular history that may be unusual because of various factors [66]. Incongruence is a recurring problem at both higher and lower phylogenetic levels [67-70]. As noted by many authors, a broad approach to illuminating and reconciling this incongruence is to analyze other genetic evidence, such as that provided by nuclear DNA.

### Mitogenome organization

We newly determined the complete (or nearly complete) L-strand nucleotide sequences for mitogenomes of the 33 lophiiform species during this study and the sequences have been deposited in DDBJ/EMBL/GenBank under the accession numbers of AB282826-56 and AP005977-8 (Table 2). For *Brachionichthys hirsutus *(AB282832), however, we were unable to sequence a region spanning from tRNA-Gly to the control region (approximately 40% of the complete sequence) owing to technical difficulties and degradation of the tissues. The genome contents include two rRNA, 22 tRNA, and 13 protein-coding genes, plus the putative control region(s), as found in other vertebrates, and most of the genes are encoded on the H-strand, except for the ND6 and eight tRNA genes.

The gene arrangements of the 33 species are identical to those of typical vertebrates, except for three species in two different suborders, a tetrabrachiid *Tetrabrachium ocellatum *(Antennarioidei) and two ceratiids, *Ceratias uranoscopus *and *Cryptopsaras couesii *(Ceratioidei), in which significant numbers of tRNA genes and the control regions in the latter two taxa are translocated from the typical vertebrate positions. Also, unlike typical vertebrates, 17 examples of relatively long non-coding sequences (>100 bp) other than the control regions occur in 12 ceratioid species in five families (Caulophrynidae, Melanocetidae, Oneirodidae, Gigantactinidae, Linophrynidae; Table 5). Among these 17 examples, insertion sequences between the ATPase 6 and COIII genes (118-682 bp; the two genes located adjacent to each other without insertions in most vertebrates) were observed in six species in four families (Caulophrynidae, Melanocetidae, Oneirodidae, Linophrynidae), while 11 other sequences were restricted to either all or some member(s) of single families (Oneirodidae, Gigantactinidae, Linophrynidae).

**Table 5:** Patterns of intergenic non-coding sequences (≥ 100 bp) and their lengths (bp) in 23 oneiroid species

Family	Species	Insertion (bp)
Caulophrynidae	*Caulophryne jordani*	A6/C3 (131)
	*C. pelagica*	A6/C3 (118)
Neoceratiidae	*Neoceratias spinifer*	--
Melanocetidae	*Melanocetus murrayi*	A6/C3 (332)
	*M. johnsonii*	A6/C3 (406)
Himantolophidae	*Himantolophus albinares*	--
	*H. groenlandicus*	--
Diceratiidae	*Bufoceratias thele*	--
	*Diceratias pileatus*	--
Oneirodidae	*Oneirodes thompsoni*	Met/N2 (111); A6/C3 (473); Glu/CB (257)
	*Puck pinnata*	Ala/Asn (255); Tyr/C1 (314); N6/Glu (489)
	*Chaenophryne melanorhabdus*	Lys/A8 (445)
	*Bertella idiomorpha*	--
	*Lasiognathus *sp.	--
Thaumatichthyidae	*Thaumatichthys pagidostomus*	--
Centrophrynidae	*Centrophryne spinulosus*	--
Ceratiidae	*Ceratias uranoscopus*	--
	*Cryptopsaras couesii*	--
Gigantactinidae	*Gigantactis vanhoeffeni*	Ser/Leu (593)
	*Rhynchactis macrothrix*	Ler/N5 (268)
Linophrynidae	*Linophryne bicornis*	N6/Glu (186)
	*Acentrophryne dolichonema*	A6/C3 (682); N6/Glu (186)
	*Haplophryne mollis*	N6/Glu (144)

Abbreviation of genes: Met = tRNA-Met; N2 = NADH dehydrogenase subunit 2; Ala = tRNA-Ala; Asn = tRNA-Asn; Tyr = tRNA-Tyr; C1 = cytochrome c oxidase subunit I; Lys = tRNA-Lys; A8 = ATPase subunit 8; A6 = ATPase subunit 6; C3 = cytochrome c oxidase subunit III; Ser = tRNA-Ser (AGY); Leu = tRNA-Leu (CUN); N5 = NADH dehydrogenase subunit 5; N6 = NADH dehydrogenase subunit 6; Glu = tRNA-Glu; CB = cytochrome *b *genes

Such gene rearrangements and patterns of insertion sequences have been employed as useful phylogenetic markers in other fishes, as well as various metazoan animals, because they may represent uniquely derived characters shared by members of monophyletic groups (for reviews, see [71] but see also [72]). These genomic features also have been demonstrated to be useful in delimiting unexpected monophyletic groups in some teleosts, such as congroid eels [73] and macrouroid cods [74]. However, the distributions of these unique genomic features across ceratioid families (not within-families; Table 5) are incongruent with the inferred inter-familial relationships derived from the nucleotide sequences (see below), suggesting either independent acquisitions or a single gain followed by independent losses of such unique features in a parsimony framework. Details of the gene rearrangements and patterns of insertion sequences in the Ceratioidei will be discussed elsewhere.

### Monophyly and phylogenetic position of the Lophiiformes

**Figure 5:**
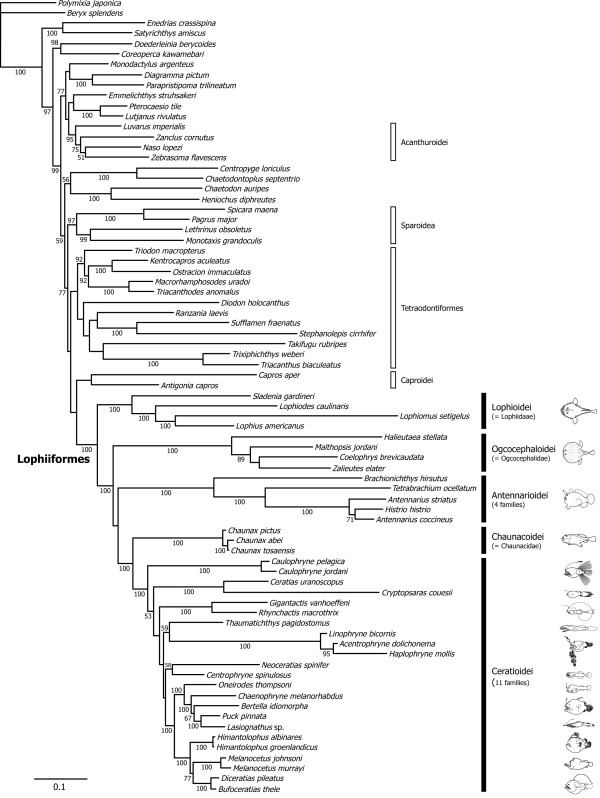
**The best-scoring maximum likelihood (ML) tree derived from 12_n_3_r_RT_n _dataset**. Numerals beside internal branches indicate bootstrap probabilities ≥50% based on 500 replicates. Scale indicates expected number of substitutions per site. Extremely long branch from *Tetrabrachium ocellatum *is shortened to one third of the original length.

Our taxon sampling assumes the percomorph Lophiiformes (not paracanthopterygian Lophiiformes as advocated by Patterson and Rosen [10]; Rosen and Patterson [7]) and the datasets comprise 44 whole mitogenome sequences used in Yamanoue et al. [27] plus those sequences from the 33 lophiiforms (Table 2). To check sensitivity of additional taxon sampling from a number of the lophiiforms to the results reported in Yamanoue et al. [27], we used their pre-aligned sequences as a basis for further alignment with the 33 sequences. As expected from this multiple alignment procedure, the resulting phylogenies outside the lophiiforms (Figure 5; derived from 12_n_3_r_RT_n _dataset) are identical to those reported in Yamanoue et al. [27] and the order Lophiiformes is confidently recovered as a monophyletic group with 100% bootstrap probabilities (BPs) in all datasets. Pietsch and Orr [33] stated that a monophyletic origin of the Lophiiformes seems certain based on six morphologically complex synapomorphic features [1-3,28] and this study is the first convincing demonstration of monophyly of the Lophiiformes based on molecular data from all the currently-recognized five suborders and appropriate taxonomic representation from outgroups in a molecular phylogenetic context.

Concerning the sister-group relationships of the Lophiiformes, no morphological study has provided a view that departs significantly from the previous paracanthopterygian notion advocated by Rosen and Patterson [7] and subsequently modified by Patterson and Rosen [10]. Both mitogenomic [25] and nuclear gene [23] phylogenies, however, have convincingly demonstrated a percomorph relationship for the Lophiiformes and nullified the hypothesis of common ancestry with the Batrachoidiformes. In fact, use of the two whole mitogenome sequences from the Batrachoidiformes as only outgroups to root the lophiiform phylogenies disrupted the monophyletic Antennarioidei at the most basal position (as in Shedlock et al. [29]), followed by divergence of the Lophioidei, Ogcocephaloidei, and a clade comprising the Chaunacoidei and Ceratioidei at the top of the tree (results not shown). These subordinal relationships, particularly the non-monophyletic and most basal position of the Antennarioidei, are similar to those reported by Shedlock et al. [29] who used the batrachoidiform sequence as an only outgroup to root their tree.

We therefore excluded those two batrachoidiform sequences in the present study, thereby revealing a sister-group relationship either with the Caproidei alone (12_n_3_r_RT_n _and 123_n_RT_n _datasets) or with the Caproidei plus Tetraodontiformes (12_n_RT_n _dataset), as shown also by Yamanoue et al. [27]. Nevertheless all nodal support values for these relationships were less than 50% bootstrap probabilities (BPs) and addition of unsampled members of the Percoidei (particularly putative members of Clade H in Kawahara et al. [75]; Yagishita et al. [76]) may eventually alter this picture of sister-group relationship of the Lophiiformes. Recently Li et al. [23] used 10 nuclear genes to analyze higher-level relationships of the actinopterygians and the only included lophiiform (a lophiid *Lophius gastrophysus*) was recovered as a sister species of two tetraodontiforms (*Takifugu rubripes *and *Tetraodon nigroviridis*). Although their dataset did not include a caproid sequence, it does appear from these and the other studies mentioned above that the tetraodontiforms are close relatives of the lophiiforms, within the Percomorpha.

### Monophyly and interrelationships of the five suborders

**Figure 6:**
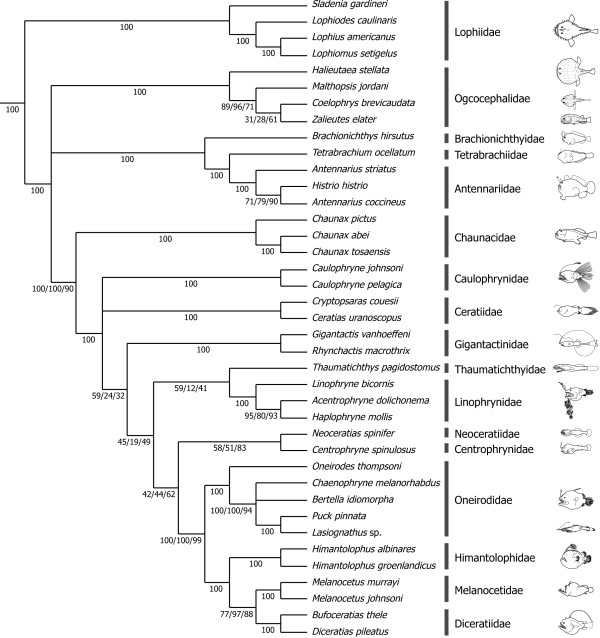
**A strict consensus of the three best-scoring maximum likelihood (ML) trees**. The strict consensus trees are derived from the three datasets that treat third codon positions differently (12_n_3_r_RT_n_, 123_n_RT_n_, 12_n_RT_n_). *Lasiognathus *sp. was considered as a member of the Oneirodidae because it is deeply nested within the family and monophyly of the traditional Thaumatichthyidae (*Thaumatichthys *and *Lasiognathus*) is confidently rejected by AU test (diff -ln *L *= 500.1; *P *> 0.0000).

The mitogenomic data strongly support monophyly for each of the five suborders, the most basal position of the Lophioidei, and monophyly of a clade comprising the rest of the four suborders (Ogcocephaloidei, Antennarioidei, Chaunacoidei and Ceratioidei) with 100% BPs (Figures 5, 6) in all datasets. The recent morphological study of Pietsch and Orr [33] also recovered monophyly of the latter clade (and the resulting most basal position of the Lophioidei) with six unambiguous synapomorphies (their characters 27, 41, 54, 70, 82 and 83). Thus this pattern of the basal divergence within the Lophiiformes (Figures 5, 6) is supported by two different lines of evidence and seems to reflect the true phylogeny.

Within a clade comprising the above four suborders, a sister-group relationship between the Chaunacoidei and Ceratioidei is consistently recovered in all datasets with high BPs (90-100%; Figure 6). Phylogenetic positions of the rest of the two suborders (Ogcocephaloidei and Antennarioidei), on the other hand, are quite ambiguous and three alternative hypotheses of relationships among three lineages (Ogcocephaloidei, Antennarioidei, and Chaunacoidei plus Ceratioidei) are almost equally likely in a statistical sense (AU test, *P *= 0.520-0.589; Table 6). Significantly, when monophyly of the Chaunacoidei plus Ceratioidei is not constrained in the statistical comparisons, all of the 12 alternative relationships are confidently rejected by AU tests (*P *= 0.000-0.030; the bottom 12 rows in Table 6), which include the morphology-based hypotheses [3,33](*P *= 0.002). Therefore the Chaunacoidei is most likely to represent the sister-group of the Ceratioidei in a mitogenomic context.

**Table 6:** Statistical comparisons among 15 alternative tree topologies of the four more derived suborders using AU test

Tree^a^	Diff -ln *L*	***P***
(Og,(An,(Ch,Ce))) ^b^	0.0	0.589
((Og,An)(Ch,Ce)) ^c^	0.0	0.577
(An,(Og,(Ch,Ce)))	0.5	0.520
(Og,(Ce,(An,Ch)))	22.4	0.030
(Og,(Ch,(An,Ce)))	27.5	0.006
(An,(Ce,(Ch,Og)))	42.5	0.015
(An,(Ch,(Og,Ce))) ^d^	43.8	0.002
((An,Ch)(Og,Ce))	47.9	0.000
(Ce,(Og,(An,Ch)))	48.9	0.000
((An,Ce)(Ch,Og))	49.7	0.002
(Ch,(Og,(An,Ce)))	50.4	0.000
(Ch,(Ce,(An,Og)))	53.2	0.004
(Ce,(Ch,(An,Og)))	54.2	0.008
(Ce,(An,(Og,Ch)))	54.6	0.000
(Ch,(An,(Og,Ce)))	55.1	0.002

^a ^Ogcocephaloidei (Og); Antennarioidei (An); Chaunacoidei (Ch); Ceratioidei (Ce). The most basal Lophioidei was excluded from the comparisons

^b ^The best-scoring ML tree derived from 12_n_3_r_RT_n _(Figure 5) and 12_n_RT_n _datasets.

^c ^The best-scoring ML tree derived from 123_n_RT_n _dataset.

^d ^Morphology-based hypothesis [3]

We acknowledge, however, that no morphological data supports a sister-group relationship between the Chaunacoidei and Ceratioidei ([33] but see [32]). Instead, morphological data have indicated monophyly of the Ogcocephaloidei plus Ceratioidei with relatively strong statistical support (BS = 94%; Bremer index = 4; see [33]) with the following three unambiguous synapomorphies: 1) the first epibranchial is simple and without ligamentous connection to the second epibranchial (character 43); 2) the third cephalic dorsal-fin spine and pterygiophore are absent (character 60); and 3) the posttemporal is fused to the cranium (character 63). However, all of these characters appear in the Ogcocephaloidei and Ceratioidei to represent simplified or reductive trends, which are perhaps more likely to have occurred convergently, and the resulting homoplasy may undermine the robustness of the phylogenetic hypotheses based on morphology [77]. Future evaluation of homology of these anatomical features, exploration of new morphological characters, and addition of molecular data from other genes may help resolve the conflict between these two different sources of phylogenetic information (for related discussion on the relationships within the Ceratioidei, see below).

### Lophiid relationships

The Lophioidei contains a single family, the Lophiidae, with 25 species distributed among four genera [78] (Table 1). Caruso [30] presented the first cladogram of lophiid genera based on 19 morphological characters (Figure 4C), of which 12 showed derived states shared by two or three genera. The reconstructed cladogram indicated the most basal position of *Sladenia*, followed by the divergence of *Lophiodes *and *Lophiomus *plus *Lophius *in sequential step-wise fashion, relationships that are fully congruent with the mitogenomic phylogenies, with all internal branches of the latter supported by 100% BPs (Figures 5, 6).

### Antennaroid relationships

The Antennarioidei contains four families with 53 species distributed among 17 genera (Table 1). Pietsch and Grobecker [3] presented a cladogram of familial relationships of the suborder based on seven synapomorphies (Figure 4D), in which the Brachionichthyidae occupies the most basal position, followed by the divergence of Lophichthyidae, with Tetrabrachiidae and Antennariidae forming a sister-group at the top of the tree [3]. Although we were unable to collect tissue samples from the only member of the Lophichthyidae (*Lophichthys boschmai*), the mitogenomic tree is completely congruent with the morphology-based phylogeny (Figures 5, 6).

Within the Antennariidae, *Antennarius striatus *is recovered as the sister of a terminal clade that includes *Histrio histrio *and *A. coccineus*, thus rendering *Antennarius *paraphyletic. The Antennariidae is by far the largest family of the suborder, including 45 species in 12 genera, of which only three species and two genera are included here. While our coverage of the Antennariidae is poor, an on-going molecular study by one of us (RJA), based on both mitochondrial and nuclear genes and considerably more taxa (25 species and 10 genera), also results in paraphyly for *Antennarius*. Thus, more extensive taxon sampling within *Antennarius *as well as within other antennariid genera is not likely to alter the topology shown here.

### Ogcocephaloid relationships

The Ogcocephaloidei contains a single family with 68 species distributed among 10 genera (Table 1). Endo and Shinohara [31], while describing a new species of the genus *Coelophrys*, cladistically analyzed nine morphological characters (all previously used in [79]) from nine of the 10 genera. As expected from such a small number of characters, resolution of the resulting cladogram was poor at the two most basal nodes (Figure 4E) and *Coelophrys *- an unusually globose genus among the typically dorsoventrally flattened ogcocephaloids - was placed at the top of the tree (Figure 4E). The placement of *Coelophrys *and the more basal *Halieutaea *in the cladogram (Figure 4E) agree with the mitogenomic phylogenies (Figures 5, 6), but the placement of *Malthopsis *and *Zalieutes *do not. A statistical test finds no significant difference between the morphological cladogram (Figure 4E) and the mitogenomic phylogeny (Figure 5) (AU test, *P *= 0.182), perhaps owing to the poor resolution of the morphological cladogram and low taxon sampling in the molecular phylogenies. Again more extensive taxon sampling will be required to obtain a better picture of their relationships.

### Chaunacoid relationships

The Chaunacoidei contains a single family with about 14 species divided between two genera [2] (Table 1). While we successfully obtained tissue samples from three species of the more common *Chaunax*, those from the rare genus *Chaunacops *were unavailable. Thus we are unable to evaluate monophyly for each of the two genera and to investigate their relationships. There is no phylogenetic hypothesis for chaunacoids at present.

### Ceratioid relationships

The Ceratioidei contains 11 families with 160 species distributed among 35 genera [2] (Table 1). The first attempt to resolve relationships among ceratioid taxa after the advent of cladistic method [9] was that of Bertelsen [32]. He admitted, however, that most of the derived osteological characters shared by two or more families are reductive states or loss of parts, and similarities among such characters may in many cases represent convergent development. Nevertheless, Bertelsen [32] presented a cladogram of the ceratioid taxa (Figure 4F), stating that the tree should be regarded only as "a very schematic compilation of expressed view." He concluded that future studies on additional characters and as yet unknown taxa may bring answers to at least some of the many questions about their phylogenetic relationships.

More recently, Pietsch and Orr [33], with the advantage of more than 20 years of additional accumulated data since Bertelsen's attempt [32], coupled with a re-examination of all previously identified characters and analyses of new characters, presented the first computer-assisted cladistic analysis of relationships of ceratioid families and genera (Figure 4G). In that study, Pietsch and Orr [33] showed two trees: one based on 71 morphological characters applicable to metamorphosed females (Figure 4G), and another one based on 17 morphological characters applicable to metamorphosed males and larvae, in addition to the 71 characters extracted from females, for a total of 88 characters. The latter tree was poorly resolved and Pietsch and Orr [33] thus considered the former as the best estimate of relationships.

Our dataset includes 23 species in 20 genera from all 11 ceratioid families. Our preferred dataset (12_n_3_r_RT_n_: RY-coding) reproduces the most basal Caulophrynidae, followed by divergence of the Ceratiidae, Gigantactinidae, Thaumatichthyidae plus Linophrynidae, Neoceratiidae plus Centrophrynidae, Oneirodidae (including *Lasiognathus*; see below), Himantolophidae, and Melanocetidae plus Diceratiidae at the top of the tree in sequential step-wise manner (Figure 5). More basal relationships among the seven families up to a clade comprising the Neoceratiidae plus Centrophrynidae are poorly resolved, with all internal branches supported by <60% BPs. Different treatments of the 3rd codon positions even yield different tree topologies, collapsing the most basal clade within the Ceratioidei in a strict consensus tree (Figure 6).

Interrelationships among the most apical four families (Oneirodidae, Himantolophidae, Melanocetidae, and Diceratiidae), on the other hand, are more robust with all internal branches supported by 99-100% BPs except for a sister-group relationship between the Melanocetidae and Diceratiidae (77-97% BPs; Figure 6). Shedlock et al. [29] also recovered an identical tree topology among the first three families (Oneirodidae, Himantolophidae, Melanocetidae) based on short sequences from the mitochondrial 16S rRNA gene, although their dataset lacked a member of the Diceratiidae. *Lasiognathus *(Figure 2H), long placed in the Thaumatichthyidae (Figure 2I), is here deeply nested within the Oneirodidae, and shown as the sister species of the derived oneirodid *Puck pinnata *at the top of the clade with 100% BPs (Figures 5, 6). The placement of *Lasiognathus *and *Thaumatichthys *in separate families was considered by Bertelsen and Struhsaker [80] who compared the osteology and pointed out that *Lasiognathus *appears more closely related to the Oneirodidae, but they chose to separate the two genera into different families. As expected from the most derived position of *Lasiognathus *within the oneirodids with the highest BPs (100%), monophyly of the traditional Thaumatichthyidae (including *Lasiognathus*) is confidently rejected by AU test (diff -ln *L *= 500.1; *P *< 0.0000).

### Incongruence between morphology and molecule

As pointed out by Pietsch and Orr [33], who compared their tree with the unpublished molecular phylogeny provided by M.M. (referred to as Miya unpubl. data), the morphological hypothesis (Figure 4G) bears very little resemblance to the mitogenomic phylogenies (Figures 5, 6). Statistical differences between the constrained and unconstrained ML trees are so large (diff -ln *L *= 793.9, *P *< 0.0000 for [32]; diff -ln *L *= 1308.8, *P *< 0.0000 for [33]) that we are unable to reconcile these competing hypotheses. In fact, among the clades that differ between the two analyses, non-homoplastic morphological characters support only one clade (Himantolophidae, Diceratiidae, and Melanocetidae) and that with only a single character (8: the condition of the ventromedial extensions of the frontals).

Such remarkable incongruence between morphological and molecular hypotheses of ceratioid relationships requires an explanation. Although additional sequence data from other portions of the genome (e.g., nuclear genes) should be analyzed to confirm molecular conclusions [67,69], Hedges and Sibley [81] argued that, in such cases of incongruence, morphological evidence should also be reevaluated. Following Hedges and Sibley's argument [81] and Bertelsen's empirical comments [32] that reductive states or loss of parts and similarities among such characters may in many cases represent convergent development, we have reviewed all of the 71 characters from the metamorphosed females and found the following 18 characters that are reductive, simplified, or absent for derived states (with the exception of those characters showing complete congruence with the molecular phylogenies; e.g., only autapomorphies for single families): vomerine teeth absent (character 3); parietal absent (9); pterosphenoids absent (10); endopterygoid absent (16); interopercle extremely reduced (23); rostral cartilage absent (26); maxillae considerably reduced (29); thick anterior maxillomandibular ligament very much reduced or absent (30); dentaries simple (31); first pharyngobranchial absent (39); first epibranchial absent (42); first epibranchial simple, not bearing a medial process (43); third hypobranchial absent (45); branchial teeth absent on the first three ceratobranchial (46); ninth or lower-most ray in caudal fin reduced (52); cephalic dorsa-fin spine absent (60); posttemporal is fused to the cranium (63); and pelvic bones reduced (66).

**Figure 7:**
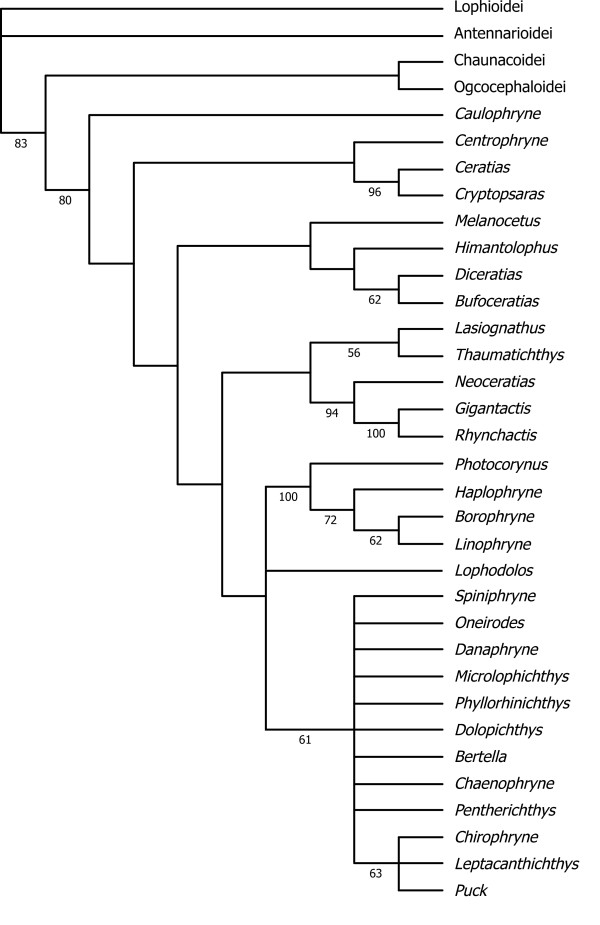
**A strict consensus of the 11 most parsimonious tree derived from maximum parsimony (MP) analysis of 53 morphological characters**. These morphological characters are applicable to the metamorphosed females only (71 characters used in Pietsch and Orr [33] minus 18 characters that are supposedly show reductive or simplified states; for details see text). The 11 MP trees had a total length of 100, a consistency index of 0.610, and a retention index of 0.835.

Assuming that all or some of these 18 reductive or simplified morphological characters likely represent homoplasy, we excluded them from the original dataset and the reduced dataset was subjected to maximum parsimony (MP) analysis, similar to that conducted by Pietsch and Orr [33]. The MP analysis produced 11 equally most parsimonious trees, with a total length of 100, a consistency index of 0.610, and a retention index of 0.835, a strict consensus shown in Figure 4. The resulting MP tree exhibits some important similarities with the molecular phylogenies that are not evident in the trees of Pietsch and Orr [33]. For example, the Caulophrynidae is placed as the most basal lineage within the Ceratioidei in the revised cladogram (Figure 7). Pietsch and Orr [33] were surprised with the derived position of the Caulophrynidae in their cladogram (Figure 4G) in light of Bertelsen's view [32,82] that the absence of an escal light organ in all life-history stages of the family is not due to secondary loss. Bertelsen's opinion [32,82] was reinforced by ontogenetic information from other characters, such as the apparent absence of sexual dimorphism in rudiments of the illicium and the absence of a distal swelling of the illicial rudiments. Our preferred mitochondrial dataset (12_n_3_r_RT_n_: RY-coding) supports the most basal position of Caulophrynidae within the Ceratioidei (and monophyly of the rest of the families to the exclusion of the Caulophrynidae), although statistical support is not convincing (53% BP in Figure 5).

The revised cladogram (Figure 7) also recovers a monophyletic group comprising the Himantolophidae, Melanocetidae and Diceratiidae that is supported by 100% BPs (Figures 5, 6). Pietsch and Orr [33] observed that these three families uniquely share a single non-homoplastic morphological character (ventromedial extensions of the frontal that make no contact with the parasphenoid). In addition to these ceratioid relationships, monophyly of the Ogcocephaloidei + Ceratioidei is collapsed to form a trichotomy of these two suborders plus Chaunacoidei. Thus simple exclusion of reduced or simplified characters from the morphological dataset yields a tree that can be better reconciled with the molecular phylogenies (Figures 5, 6). However, simply deleting all reductive characters may also be misleading, by running the risk of rejecting informative characters. Homology of reductive morphological characters is commonly evaluated by ontogenetic analysis, but in the case of ceratioids, very little ontogenetic material is available for analysis [2,33]. Considerably more work will be needed to further reconcile these competing phylogenetic hypotheses.

### Evolution of male sexual parasitism

**Figure 8:**
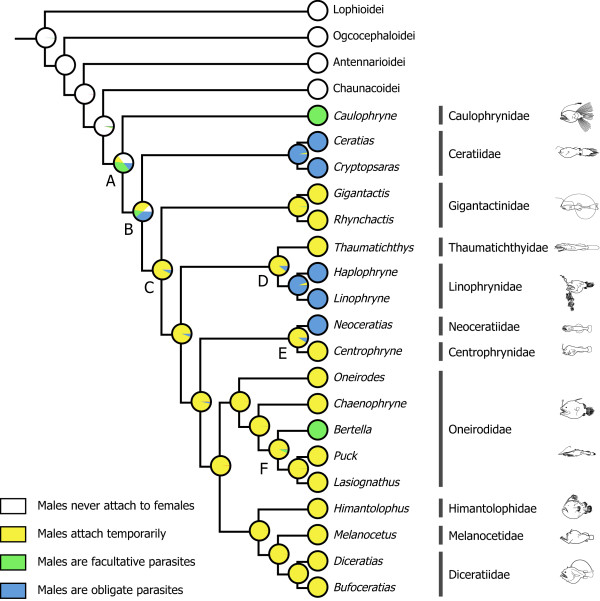
**Maximum likelihood reconstruction of the male sexual parasitism in ceratioid anglerfishes**. Four discrete character states were assigned to each terminal and ancestral character states were reconstructed on the ML tree (Figure 5) under an ML optimality criterion using Mesquite ver. 2.6 [56].

The maximum likelihood (ML) reconstruction of the four reproductive modes in ceratioid males on the mitogenomic phylogenies reveals that character states at the two ancestral, most basal nodes (A and B in Figure 8), are equivocal. The character states 0 (males never attach to females) and 2 (males are facultative parasites) are almost equally likely at node A (*P*_0 _= 0.356; *P*_2 _= 0.381), as are the character states 1 (males attach temporarily) and 3 (males are obligate parasites) at node B (*P*_1 _= 0.348; *P*_3 _= 0.390). Thus we are unable to determine ancestral states of facultative and obligate parasitic males in the Caulophrynidae (node A) and Ceratiidae (node B), respectively (Figure 8). With the exception of these two basal families, evolutionary origins of parasitic males are unequivocally reconstructed on the mitogenomic phylogenies in more derived clades above node C (Figure 8). For example, precursors of those taxa with obligate (Linophrynidae and Neoceratiidae) and facultative (the oneirodid *Bertella*) parasitic males are reconstructed as the temporal attachment of males at nodes D, E, and F with high probabilities (*P*_1 _= 0.893-0.995; Figure 8). On the basis of their morphological cladogram, Pietsch and Orr [33] stated that whether facultative parasitism and temporary attachment of males to females are precursors to obligate parasitism, or the former are more derived states of the latter, remains unknown. Our ML reconstruction strongly suggests that temporary attachment of males to females is a precursor to facultative or obligate parasitism for at least three of the five cases at the family level (Figure 8).

Pietsch and Orr [33] further argued that the disjunct pattern of sexual parasitism within ceratioids appears to be the result of independent acquisition among the various lineages rather than a repeated loss of this attribute within the suborder. To support this argument, Pietsch and Orr [33] listed many differences in the precise nature of male-female attachment among the various taxa [4], to the extent of the most extreme possibility being an independent acquisition of sexual parasitism within families, such as the Ceratiidae (*Ceratias *vs. *Cryptopsaras*) and Linophrynidae (*Haplophryne *vs. *Linophryne*). If so, evolution of sexual parasitism has independently occurred as many as seven times within the suborder (= the number of green or blue circles at terminal nodes in Figure 8). Similarly, although our simple character coding does not take into account such differences in male-female attachment, our ML reconstruction suggests that acquisition of this attribute has occurred at least five times during ceratioid evolution. Shedlock et al. [29] also found a paraphyletic pattern of sexual parasitism within the suborder in their much smaller dataset and suggested that the plasticity of this unique life history trait among vertebrates is likely shaped by a dynamic relationship between localized population densities and the feasibility of maintaining mate choice at low effective population size in the expanse of the deep ocean. Of course, it may be possible that availability of more specimens from these rare organisms will shed a new light for evolution of the male sexual parasitism.

### Divergence time estimation

As Carnevale and Pietsch [34] stated, fishes of the order Lophiiformes are very rare in the fossil record and all of the recorded ages fall in the Cenozoic from 7.6 to 40 Myr ago (for details, see Table 4). Assuming a sister group relationship of the Lophiiformes and Tetraodontiformes, however, the origin of the modern Lophiiformes can be dated to the deep Mesozoic, because an articulated fossil that is convincingly assignable to the modern Tetraodontiformes was discovered from the mid Cretaceous (Cenomanian) 98 Myr ago [83]. This fossil lineage would have appeared well after the divergence of the common ancestor of the Lophiiformes and Tetraodontiformes. Fossils are useful only for minimum time constraints to estimate divergence times of the Lophiiformes, as generally acknowledged [40,84].

**Figure 9:**
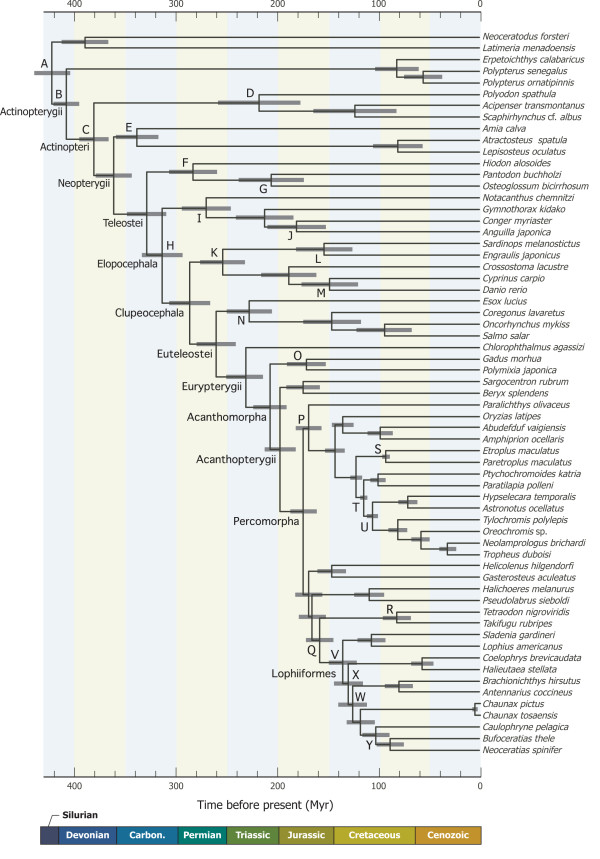
**Divergence times of ray-finned fishes**. Divergence times were estimated from the partitioned Bayesian analysis using a multidistribute program package [63]. A total of 25 nodes (A-Y) were used for time constraints (for details, see Table 4). Horizontal bars indicate 95% credible intervals of the divergence time estimation.

A relaxed molecular-clock Bayesian analysis of divergence time estimates in the present study (Figure 9), which is based on 31 time constraints (Table 4), shows excellent agreement with previous studies based on whole mitogenome sequences (Table 7). Therefore, the analysis is not sensitive to the taxon sampling strategy employed to avoid a "node density effect" (i.e., sampling a minimum number of lophiiform species[61,62]). The Lophiiformes is estimated to have diverged from an ancestral lineage of the Tetraodontiformes (the putative sister-group in the present dataset) 157 Myr ago (145-172 Myr ago; 95% credible interval) (Figure 9). Although a common ancestral lineage of the Lophiiformes has failed to leave extant lineages for about 23 Myr, it has subsequently diversified into five subordinal lineages in a relatively short time interval of 18 Myr between 117 and 135 Myr ago: a common ancestor of the order is estimated to have diverged into the Lophioidei and the rest of the four suborders 135 Myr ago (121-149 Myr ago), followed by the divergence into the Ogcocephaloidei and the rest of the three suborders 129 Myr ago (115-144 Myr ago), the Antennarioidei and the rest of the two suborders 125 Myr ago (112-140 Myr ago), and the Chaunacoidei and Ceratioidei 117 Myr ago (104-131 Myr ago). Significantly, ancestral lineages of the modern Lophiiformes have occupied various marine habitats, from relatively shallow benthic to (principally) deep bathypelagic (>1000 m deep) environments, within this short time period (18 Myr). This time period roughly corresponds to the beginning of the Gondwanian fragmentation [85,86] which, with these vicariant events, produced diversified coastal marine environments, with various niches along the continental shelves.

**Table 7:** Comparisons of divergence time estimates between the present study and previous studies

Node	This study (Figure 9)	**Azuma et al. **[40]^a^	**Setiamarga et al. **[84]
Sarcopterygii vs. Actinopterygii	421 (403-439)	429 (417-449)	428 (419-442)
Teleostei vs. Neopterygii	360 (340-376)	365 (348-378)	364 (346-378)
Euteleostei vs. Otocephala	285 (265-305)	288 (268-307)	315 (270-363)
*Cyprinus *vs. *Danio*	148 (121-176)	147 (120-174)	153 (125-183)
Acanthopterygii vs. Paracanthopterygii	206 (190-224)	207 (190-224)	209 (191-225)
Percomorpha vs. Berycomorpha	196 (182-212)	198 (183-215)	200 (185-217)
*Oryzias *vs. Tetraodontiformes	174 (161-187)	176 (163-191)	180 (166-195)
*Oryzias *vs. Cichlidae	143 (134-153)	152 (141-165)	150 (139-161)
*Gasterosteus *vs. Tetraodontidae	169 (156-183)	170 (156-185)	173 (159-189)
*Takifugu *vs. *Tetraodon*	81 (68-96)	78 (65-93)	78 (63-93)

^a ^Estimated with biogeography-based time constraints on cichlid divergence

Unique among principally bathypelagic ceratioids are three species of the genus *Thaumatichthys *(Thaumatichthyidae; Figure 2I) that are abyssal-benthic, presumably staying in deep-sea bottom (> 1000 m) and luring prey items with esca "inside the mouth" [80]. If this unusual life style had been attained concurrently in the origin of the common ancestor of *Thaumatichthys*, it took about 33 Myr after leaving the bottom of the sea around the continental shelves and subsequently returning to that unique benthic life style at greater depths.

### Patterns and rates of diversification

**Figure 10:**
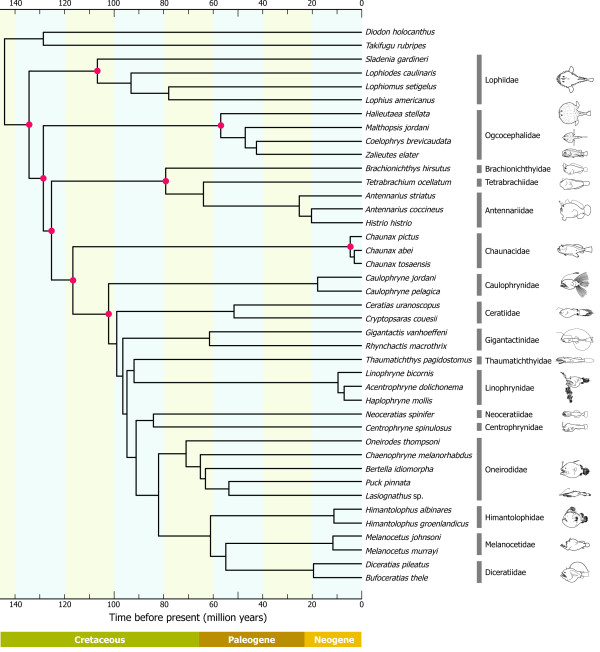
**Divergence times of the 39 species of the Lophiiformes**. Divergence times were estimated from the partitioned Bayesian analysis using a multidistribute program package [63]. A total of nine nodes (filled circles) were used for fixed time constraints.

The resulting time tree of the Lophiiformes (Figure 10) allows us to provide some insights into the patterns and rates of diversification across the order. Although incomplete taxon sampling from some of the suborders (Ogcocephaloidei, Antennarioidei, Chaunacoidei) prohibited rigorous evaluation of the patterns of diversification across the Lophiiformes, there are remarkable differences between diversification patterns in the Lophioidei (= Lophiidae) and Ceratioidei, for which we successfully sampled all of the genera and families (Figure 10). An ancestral lineage of the Lophioidei began to diversify 109 Myr ago, leaving only four modern genera during a period of about 27 Myr. Almost concurrently, an ancestral lineage of the Ceratioidei began to diversify 103 Myr ago, leaving as many as eight modern families plus a common ancestor of the three more derived families (Himantolophidae, Melanocetidae, Diceratiidae) during a period of about 20 Myr, suggesting rapid morphological radiations during an early phase of ceratioid evolution at bathypelagic depths. Such rapid familial radiations and the resulting short internal branches may render the phylogenetic analysis difficult to resolve the basal relationships (Figure 6).

Per-clade net diversification rates based on stem-node ages and current species diversity, on the other hand, can be compared across all subordinal lineages. Accordingly we estimated net diversification rates (*b *- *d*, where *b *is the speciation rate and *d *is the extinction rate) per clade, under the lowest extinction rate (*d:b *= 0) and under an extremely high relative extinction rate (*d:b *= 0.95) for each clade (Table 8). With a known diversity of 361 modern species (Table 1) and an estimated basal split at 157 Myr ago (Figure 9), the Lophiiformes exhibit an average net diversification rate of 0.0368 event per lineage per million years under *d:b *= 0 and 0.0181 event per lineage per million years under *d:b *= 0.95. As expected from differences in the current diversity and similar stem node ages, the Ceratioidei exhibits remarkably higher net diversification rates of 0.0434 event per lineage per million years under *d:b *= 0 and 0.0188 event per lineage per million years under *d:b *= 0.95 (Table 8) than those of the rest of the four suborders (0.0231-0.0334 under *d:b *= 0; 0.0045-0.0115 under *d:b *= 0.95). With the acquisition of novel features, such as male dwarfism, bioluminescent lures, and unique reproductive modes, it appears that a ceratioid invasion of a largely unexploited bathypelagic zone allowed for explosive diversification in a relatively brief period.

**Table 8:** Per-clade net diversification rates (events per lineage per Myr) for the five suborders of the Lophiiformes

Suborder	Number of species	Divergence time (Myr ago)	*r*_0_	*r*_0.95_
Lophioidei	25	134.7	0.0239	0.0059
Ogcocephalidae	54	129.2	0.0309	0.0100
Antennarioidei	66	125.6	0.0334	0.0115
Chaunacoidei	15	117.2	0.0231	0.0045
Ceratioidei	161	117.2	0.0434	0.0188

The rates were calculated using a Magallón and Sanderson's method-of-moment estimator [64] assuming two extreme extinction rates (*ε*) of 0 and 0.95

## Conclusions

The mitogenomic analyses demonstrated previously unappreciated phylogenetic relationships among the lophiiform suborders and deep-sea ceratioid familes. Although the latter relationships cannot be reconciled with the earlier hypotheses based on morphology, we found that simple exclusion of the reductive or simplified characters can alleviate some of the conflict. Reconstruction of the male reproductive modes of the ceratioids on the resultant phylogeny revealed complex evolutionary patterns of the sexual parasitism in males. A relaxed molecular-clock Bayesian analysis of the divergence times suggests that all of the subordinal diversifications have occurred during a relatively short time period between 100 and 130 Myr ago (early to mid Cretaceous). Comparisons of per-clade net diversification rates among the five lophiiform suborders suggest that the acquisition of novel features, such as male dwarfism, bioluminescent lures, and unique reproductive modes allowed the deep-sea ceratioids to diversify rapidly in a largely unexploited, food-poor bathypelagic zone (200-2000 m depth) relative to the other lophiiforms occurring in shallow coastal areas along continental shelves.

## Authors' contributions

MM, TWP and MN designed this study. MM, TWP, TPS, HCH, MS and MY mainly collected the specimens. MM and TPS carried out the molecular work and analyzed the data. MM drafted the original manuscript and TWP, JWO, RJA, AMS, HCH, MS, and MY contributed to its improvement. All authors read and approved the final manuscript.
